# Poly ****β****-Hydroxybutyrate Production by *Bacillus subtilis* NG220 Using Sugar Industry Waste Water

**DOI:** 10.1155/2013/952641

**Published:** 2013-08-21

**Authors:** Gulab Singh, Anish Kumari, Arpana Mittal, Anita Yadav, Neeraj K. Aggarwal

**Affiliations:** ^1^Department of Microbiology, Kurukshetra University, Kurukshetra 136119, India; ^2^Department of Biotechnology, Kurukshetra University, Kurukshetra 136119, India

## Abstract

The production of poly **β**-hydroxybutyrate (PHB) by *Bacillus subtilis* NG220 was observed utilizing the sugar industry waste water supplemented with various carbon and nitrogen sources. At a growth rate of 0.14 g h^−1^ L^−1^, using sugar industry waste water was supplemented with maltose (1% w/v) and ammonium sulphate (1% w/v); the isolate produced 5.297 g/L of poly **β**-hydroxybutyrate accumulating 51.8% (w/w) of biomass. The chemical nature of the polymer was confirmed with nuclear magnetic resonance, Fourier transform infrared, and GC-MS spectroscopy whereas thermal properties were monitored with differential scanning calorimetry. In biodegradability study, when PHB film of the polymer (made by traditional solvent casting technique) was subjected to degradation in various natural habitats like soil, compost, and industrial sludge, it was completely degraded after 30 days in the compost having 25% (w/w) moisture. So, the present study gives insight into dual benefits of conversion of a waste material into value added product, PHB, and waste management.

## 1. Introduction

The diverse group of chemicals used in making of plastic is highly known to be highly toxic and poses a serious threat to biosphere. These substances besides hitting hard to ecosystem can also cause an array of problems like birth defects, cancer, damage of nervous, and immune systems. Polypropylene—the key ingredient for plastic—is a petroleum product and becomes increasingly expensive. With increasing concern for the environment, biosynthetic and biodegradable biopolymers have attracted great interest because of their excellent biodegradability and being environmentally benign and sustainable.

The PHAs (hydroxyalkanoic acid) are synthesised by wide range of microorganisms as intracellular carbon and energy reserve material under nutrient limiting conditions [1]. The first identified PHA, poly-*β*-hydroxybutyrate (PHB) from *Bacillus megaterium*, is drawing much attention due to having physical properties similar to petroplastic polypropylene [2] with advantage of being completely biodegradable (into water, carbon dioxide, and methane under anaerobic conditions) by microorganisms in natural environments. The high production cost of PHB can be curtailed by strain development, improving fermentation and separation processes, and using inexpensive carbon source. In PHB production, about 40% of the total production cost is accounted for raw material, and thus, the use of inexpensive carbon source or even waste organic materials could be highly significant.

Annually, the million tons of organic wastes are produced from the wide array of industries and in this context, contribution from sugar industries is worst in terms of effluent production—out of all the major sugar manufacturing units, that is, mill house, process house, boiler house, alcohol producing unit, and distilleries [3]. The quantity of waste water generated ever increasing with sugar production—where a typical sugar mills consume around 2000 litres of water and generate about 1000 litres of waste water per ton of cane—crushed. The waste water contains floor washing waste water and condensate water, sugarcane juice, syrup and molasses, and so forth. The sugar mill effluent has a high biochemical oxygen demand (BOD) and if discharged untreated, increased BOD effects aquatic ecosystem and has ultimate impact on human health.

The sugar industries need spending lots of money on the treatment of waste water treatment to adhere to regulatory standards.

Thus the two different problems, namely, the pollution due to synthetic plastic and generation of organic rich waste from sugar industries could uniquely addressed by producing the bioplastic using the organic waste as nutrient source.

However, this carbohydrate waste lacks certain mineral nutrient, particularly nitrogen in relation to the amount of oxidizable carbon present and still may require nutrient supplementation.

 Here, an attempt was made to use the sugar industry waste water after nutritive adjustment for the production of PHB using an isolate *Bacillus subtilis NG220*.

## 2. Materials and Methods

### 2.1. Bacterial Isolation and Culturing

*Bacillus subtilis* NG220 was isolated on carbon rich nutrient agar medium [(w/v) glucose 1%, beef extract 0.3%, peptone 0.5%, and sodium chloride 0.8%, agar 1.5%] from the soil sample collected from sugarcane field area. The presence of intracellular PHB granules was confirmed with the help of staining with Sudan black B and Nile blue A [4]. The culture was maintained on the nutrient agar medium (w/v) (beef extract 0.3%, peptone 0.5%, sodium chloride 0.8%, and agar 1.5%) and stored at 4°C.

### 2.2. Morphological, Biochemical and Molecular Characterisation

The isolate was morphologically characterized by observing the standard microbiological methods. The biochemical characterization of the isolate was done by series of biochemical tests including carbohydrate fermentation, H_2_S production, MR-VP test, and Catalase test. Further, for molecular characterization, the PCR amplification of 16S rDNA was done at 95°C for 5 min, 95°C for 30 sec. 52°C for 30 sec. 72°C for 1 min, and 72°C for 10 min. The universal primer 16sF-5′ AGA GTT TGA TCC TGG CTC AG 3′ and 16sR-5′ ACG GCT ACC TTG TTA CGA CTT 3′ were used. PCR product was purified by column (Merck bioscience, India) and was sequenced with AB instrument 3730XL. The obtained sequence was subjected to search for closest possible species using distance matrix based on nucleotide sequence homology (using Kimura-2 parameter) and BLAST tools available at National Centre for Biotechnology Information (NCBI). Phylogenetic analysis was done by using the Phylip package and ClustalW.

### 2.3. Preparation of Seed Inoculum

One loop full of the culture from slant was inoculated in 5 mL of sterile nutrient broth (beef extract 0.3%, peptone 0.5%, sodium chloride 0.8%). After incubation for 24 h at 30°C, 1% (v/v) of culture having 10^8^ cells/mL was aseptically transferred into 50 mL, sterile nutrient broth and incubated at 30°C for 18 h.

### 2.4. Substrate Preprocessing

Untreated sugar industry effluent was collected from the Saraswati Sugar Mills, Yamunanagar, Haryana, India, and was stored at 4°C until used. The effluent was filtered through the muslin cloth to remove the undesired solid materials. Appropriately diluted, filtered waste water was used as PHB production medium (Table 1).

### 2.5. Production, Detection, Extraction, and Purification of PHB

The PHB production was followed in 250 mL Erlenmeyer flask containing 50 mL sugar industry waste water as production medium under stationary condition of growth. After 72 h of incubation at 30°C, culture broth was centrifuged at 8000 rpm for 15 min. The portion of the palette was resuspended in 1 mL of sterile distilled water sonicated (Hielscher, Germany) in 0.2% aq. sol. of Nile blue A, air-dried the smear and observed under phase contrast microscope [4]. Remaining amount of the pellet obtained was used for extraction and recovery of PHB according to Law and Slepecky [5] with minor modifications. Briefly, the harvested cells were lyophilized and the dry cell mass weight was noted. The dry cell mass was subsequently incubated with 10 mL sodium hypochlorite at 50°C for 1 h for cells lysis. The cell extract obtained was centrifuged at 12000 rpm for 30 min, then washed sequentially with distilled water, acetone, and absolute ethanol, and dissolved in 10 mL boiling chloroform (Sigma Aldrich). After evaporation 10 mL of conc. sulphuric acid was added and placed in water bath for 10 min at 100°C to convert the PHB into crotonic acid. On cooling, absorbance was taken at 235 nm [6] using PHB (Sigma Aldrich) as standard.

### 2.6. Polymer Analysis

#### 2.6.1. NMR Analysis: ^1^H Nuclear Magnetic Resonance

 The identity of individual monomer unit was confirmed by proton nuclear magnetic resonance (^1^H-NMR) spectroscopy. ^1^H-NMR spectra of PHB sample were recorded in CDCl3 on Bruker ACF 300 spectrophotometer at 300 MHz by using “Tetramethylsilane” as internal standard [7].

#### 2.6.2. FT-IR Analyses

FT-IR analysis of the polymer sample was carried out on MB-3000, ABB FTIR spectrophotometer in the range 4000–600 cm^−1^.

#### 2.6.3. DSC Analysis

TA Q10 series instrument was used to carry out thermal analysis of PHB sample under flowing nitrogen atmosphere at a heating rate of 10°C min^−1^; *α*-alumina powder was used as reference material using aluminium sample holder for taking thermogram [8]. In order to ensure the uniformity of temperature and good reproducibility, small amount (2–5 mg) of sample was taken. To verify the reproducibility duplicate runs were adopted under the same experimental conditions.

#### 2.6.4. GC-MS Analysis

Purified polymer, prepared as described before, was dissolved in chloroform (5 mg PHB mL^−1^), and 3 *μ*L was injected into a GC-MS instrument (Model 6890; Hewlett Packard). The column and temperature profile used for GC analysis were as described by Schubert et al. [9].

### 2.7. Optimization of PHB Production

The optimization for maximum PHB production by *Bacillus subtilis* NG220 was carried out under stationary conditions of growth using sugar industry waste water as production medium. The sugar industry waste water was filtered through muslin cloth and then rough filter paper before any study to remove any type of debris or solid waste. Several cultural parameters were evaluated to determine their effect on biomass and PHB production using sugar industry waste water. The optimized value for each parameter was selected and kept constant for further experiments. The fermentation was carried out in 250 mL Erlenmeyer flask containing 50 mL sugar industry waste water as production medium, under stationary as well as shaking condition of growth. Several cultural parameters like time of incubation (24–120 h), temperature of incubation (20–50°C), supplementation of various carbon and nitrogen sources, inoculum age and size (12–42 h, 1–3%), effect of pH (4–8), and so forth, were evaluated to determine their effect on biomass accumulation and PHB production.

#### 2.7.1. Effect of Carbon and Nitrogen Source on PHB Production

Production of PHB was followed using the optimized values of various parameters obtained from different experiments as per Table 2. PHB production was carried out using production medium supplemented with different carbon sources at 1% (w/v), namely, dextrose, xylose, sucrose, rhamnose, mannitol, maltose, lactose monohydrate, mannose, galactose, starch, and raffinose pentahydrate. PHB production was also studied using production medium supplemented with various inorganic (ammonium chloride, ammonium sulphate, di-ammonium hydrogen phosphate, and ammonium di-hydrogen phosphate, ammonium per sulphate) and organic (peptone, urea, and tryptone) nitrogen sources at 1% (w/v). Further, effect of the selected carbon and nitrogen source on PHB production was investigated at their different concentrations.

### 2.8. Preparation of Polymer Blend Sheet

Conventional solvent cast technique [10] was used for preparation of polymer blend sheet. The PHB powder extracted from *Bacillus subtilis* NG220 was mixed with soluble starch in ratio of 4 : 1 (w/w) and then dissloved in 20 mL of chloroform. The solution was poured into an open flat Petri plate and allowed to evaporate slowly at room temperature. The sheet was then cut into small pieces and used for degradation studies.

### 2.9. Degradation of Polymer Sheet

Biodegradability testing was performed by soil burial method [11]. A preweighted, 30 mm pieces were buried either into 200 g of freshly collected soil sample, compost, or sludge in wide mouth glass beakers. The beakers were filled with 200 g of soil, sludge or, compost. The mouths of the beakers were kept open and incubated at room temperature up to 40 days. Different moisture content (15%, 20%, 25%, and 30%) in the samples was maintained by adding sterile water to study the effect of the moisture on degradation of PHB films. Degradation of PHB was recorded as the loss of the weight after definite period of incubation.

## 3. Results and Discussion

### 3.1. Isolation of PHB Producing Bacteria

More than 300 isolates from different ecological niches were obtained on nutrient agar medium. The simplest first line screening program for PHB producing bacteria is the use of Sudan black B staining. The isolates which were found positive for PHB granules after Staining with Sudan black B (i.e., showing dark spot inside the pink coloured cells) were further confirmed for their PHB producing potential with Nile Blue A, a more specific dye for PHB granules, in which bright orange colour fluorescence was observed under the phase contrast microscopy.

### 3.2. Identification of Isolate

The isolate NG220 used in the present study was morphologically and biochemically characterised as per given in Table 3.

**Figure 1:**
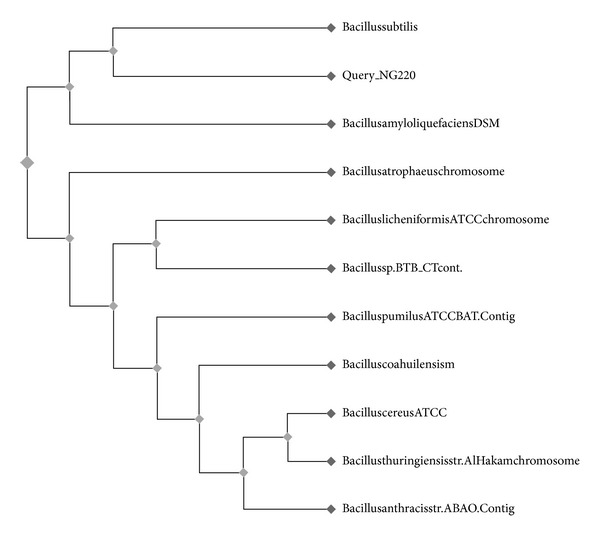
Phylogenetic tree of isolate NG220.

The isolate on the basis of their morphological and biochemical characteristics identified as *Bacillus sp.* (Bergey's Manual 9th ed.) when further characterized with 16S r-RNA genes for sequence homology (using BLAST and distance matrix based nucleotide sequence homology (Table 4)) isolate NG220 had the closest matching with the *Bacillus subtilis* (Figure 1) and was reffered to as *Bacillus subtilis* NG220. The 16S r-RNA sequence of the isolate has been submitted to the NCBI gene bank (Accession no. JQ797306).

### 3.3. Physiochemical Characterization of Sugar Industry Waste Water

The waste water had a high COD (1395 ± 317 mg/L) and BOD (1300 ± 238 mg/L). The nitrogen and phosphorous concentration in the sugar industry waste water were recorded as 11.48 ± 6.54 mg/L and 4.36 mg/L, respectively. The waste water has acidic to neutral pH and have high load of suspended solid. This waste water was used for PHB accumulation studies by the isolate with or without supplementation of different carbon and nitrogen sources.

### 3.4. Microbial Growth and PHB Production in Sugar Industry Waste Water

As such there is no report for PHB accumulation by microbial cells using sugar industry waste water. In different concentration of sugar industry waste water, growth of *Bacillus subtilis* NG220 was observed (Table 5) and the maximum growth was noted in undiluted (100% v/v) sugar industry waste water after 72 h of incubation at 30°C. Dilution of waste water not only affected the microbial growth but also PHB accumulation. This may be due to the fact that with increasing dilution of waste water the carbon and other nutrient got diluted and was not able to support microbial growth. Under unoptimized conditions *Bacillus subtilis* NG220 was observed to accumulate about 4.928 g/L of PHB after 72 h.

### 3.5. Characterisation of Extracted Polymer

#### 3.5.1. ^1^H-NMR Spectroscopy

**Figure 2:**
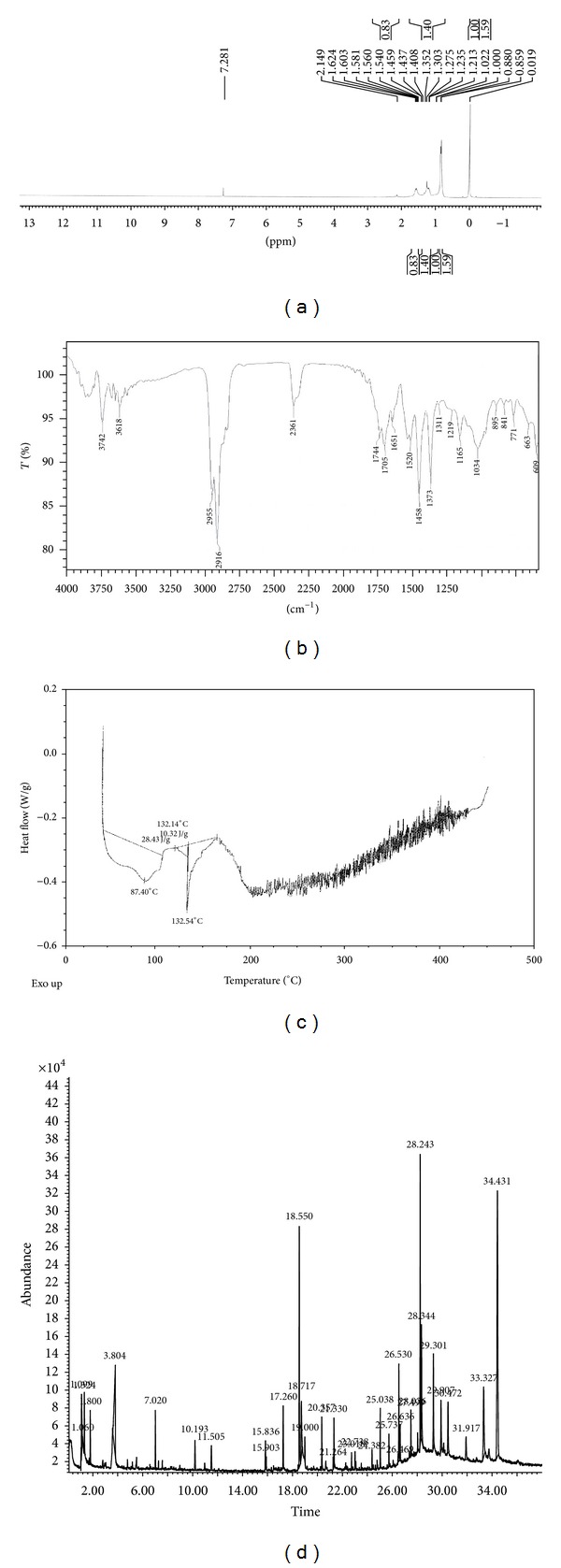
(a) NMR spectra, (b) FTIR spectra, (c) thermogram by DSC analysis, and (d) GC-MS spectra of extracted PHB polymer.

The chemical nature of extracted polymers from *Bacillus subtilis* NG220 was confirmed by ^1^H-NMR spectroscopy. The ^1^H-NMR spectra (Figure 2(a)) showed the presence of three signals, characteristic of the polymer of HB. The ^1^H-NMR spectra of polymer extracted from isolate showed a doublets at 0.85 ppm, corresponding to the methyl group (–CH3), and two multiplets at 1.23 and 1.56 ppm corresponding to methylene group (–CH2–) and methyne (–CH–) group, respectively [12]. In this study with reference PHB standard, the nature of polymer produced by the isolate was confirmed.

#### 3.5.2. Fourier Transforms Infrared Spectroscopy (FTIR)

Polymer extracted from *Bacillus subtilis* NG220 was used for recording IR spectra in the range 4000–600 cm^−1^. IR spectra (Figure 2(b)) showed two intense absorption band at 1705 and 1034 cm^−1^, specific for C=O and C–O stretching vibrations, respectively. The absorption bands at 2916 and 2955 cm^−1^ are due to C–H stretching vibrations of methyl, methylene groups. These prominent absorption bands confirm the structure of poly-*β*-hydroxybutyrate.

#### 3.5.3. DSC Analysis of PHB Polymer

The melting temperature of the extracted PHB sample obtained was determined using DSC. The thermal properties of the polymer such as the melting temperature (*T*_*m*_) are crucial for polymer processing. The melting temperature of extracted PHB (132.54°C) (Figure 2(c)) was lower as compared to that was reported in the literature (173–180°C). The melting point near to 130°C showed that the extracted PHB contained the nearly 15 mol% HV in the PHB polymer [13].

#### 3.5.4. GC-MS Analysis of Extracted PHB

In this study, the PHB was methanolysed in the presence of sulphuric acid and methanol, and the methanolysed 3HB was then analyzed by GC-MS.  Figure 2(d) showed that a common molecular fragment of the 3HB methyl ester ion chromatogram of the PHB produced. A predominant peak corresponding to the 3HB methyl ester was noted at 2.1 min, while four other small peaks were observed at 2.7, 3.3, 3.5, and 3.8 min. Of these, the peak at 3.3 min was speculated to be an impurity based on its ion fragment pattern. The retention times and ion fragment patterns of the peaks at 2.7 and 3.8 min were identical to those of the methyl esters of 3HV and 3HHx, respectively. Nevertheless, the 3HHx content was negligible in this copolymer (less than 0.1 mol%). The peak at 3.5 min also had an ion fragment pattern similar to that of the 3HHx methyl ester; however, its retention time was not identical to that of 3HHx.

### 3.6. Optimization of PHB Production

#### 3.6.1. PHB Production versus Incubation Time

**Figure 3:**
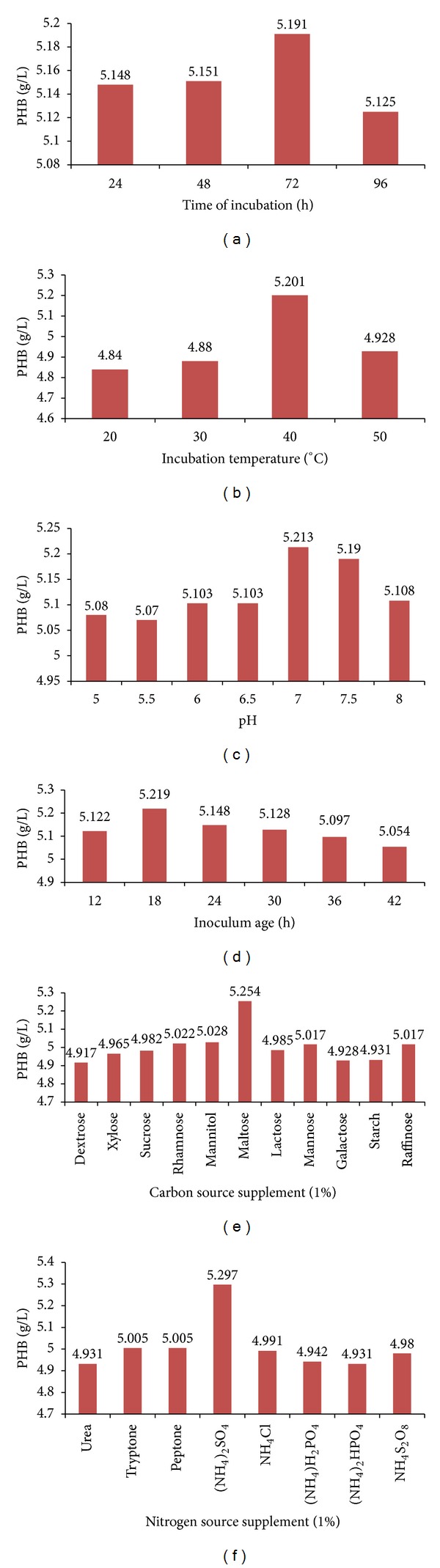
Optimization of cultural conditions for *Bacillus subtilis* NG220.

The PHB production in sugar industry waste water was followed for 96 h under stationary growth conditions. The production of PHB increased up to 72 h (5.191 g/L), and thereafter, got reduced (5.125 g/L after 96 h) (Figure 3(a)). The decrease of PHB production after 72 h might indicate that the bacteria used PHB as nutrient source. Matavulj and Molitoris [14] reported that the highest PHB level in *Agrobacterium radiobacter* was achieved during stationary growth phase after 96 h. The observation was supported by Yüksekdağ et al. [15].

#### 3.6.2. Effect of Incubation Temperature and pH on PHB Production

The maximum PHB production of 5.201 g/L was recorded at 40°C after 72 h. The increase of temperature beyond 40°C has negative impact on PHB production (Figure 3(b)). The decrease in PHB production at high temperature could be due to low PHB polymerase enzyme activity [15].

The effect of pH variations on PHB production is shown in Figure 3(c). From analysis it is clear that pH 7 was favourable for PHB production by *Bacillus subtilis* NG220 in sugar industry waste water. The current observation was in agreement with Aslim et al. [16] where the authers observed that the maximum PHB was produced (0.01 to 0.5 g/L) at pH 7 by *Rhizobium* strain grown on yeast extract mannitol broth while studying the effect of different pH on exo-polysaccharide and PHB production in two strains of *Rhizobium meliloti*. Tavernier et al. [17] reported that these two strains showed higher PHB content at pH 7.0. Shivakumar [18] reported optimum pH between 6.8 and 8.0 for PHB production by *Alcaligenes eutrophus*.

#### 3.6.3. Age of Inoculum

The maximum PHB production has been achieved after 72 h when the sugar industry waste water was inoculated with 18 h old inoculum (1% v/v), at 40°C (Figure 3(d)). Kumbhakar et al. [19] also reported that root nodule bacteria have produced maximum PHB with 12 h and 16 h old inoculum. Ramadas et al. [20] achieved the maximum PHB production by *Bacillus sphaericus* when production medium was inoculated with 16 h old inoculum.

#### 3.6.4. Effect of Different Carbon and Nitrogen Source Supplementation on PHB Production

To find the best available carbon source and its optimum concentration for maximum PHB production, commercial carbohydrates dextrose, xylose, sucrose, rhamnose, mannitol, maltose, lactose monohydrate, mannose, galactose, starch, and raffinose were tried as a sole carbon source supplement to sugar industry waste water under stationary culture conditions. From Figure 3(e) analysis it is clear that maltose supplementation to production media gave PHB yield of 5.254 g/L. Hori et al. [21] reported that PHB content in *B. megaterium* reached maximum level in a medium containing glucose as carbon source. The study conducted by Wu et al. [22] reported that *Bacillus sp.* JMa5 accumulated 25%–35%, (w/w) PHB during sucrose fermentation [23]. Working with different carbon sources in MSM broth, Khanna and Srivastava [24] observed higher PHB yield on fructose by *A. Eutrophus* [24]. Out of various organic and inorganic nitrogen sources ammonium sulphate (1% w/v) supported the maximum (5.297 g/L) PHB production followed by peptone and tryptone (Figure 3(f)). In the literature, ammonium sulphate is reported to be the best nitrogen source for PHB production in different microorganism such as *Alcaligenes eutrophus* [25], *Methylobacterium sp.* [26], and *Sinorhizobium fredii* [27]. The highest PHB was obtained in ammonium sulphate by halotolerant photosynthetic bacteria *Rhodobacter Sphaeroides* when cultivated under aerobic and dark conditions [28]. The highest level of PHB accumulation was observed in the media with protease peptone as nitrogen sources by Aslim et al. [16] in *B. subtilis 25* and in *B. megaterium 12*.

### 3.7. Evaluation of Kinetics Parameters

The evaluation of kinetic parameter for batch fermentation was studied with respect to PHB and biomass production using sugar industry waste water as production media. The *Bacillus subtilis* NG220 grew at the rate 0.141 g L^−1^ h^−1^ of production media and accumulated 51.8% of PHB at the rate of 0.0072 gg^−1^ (biomass) h^−1^. Coats et al. have reported that in some mixed cultures or activated sludge, PHA production could be as high as over 50% of the cell dry weight (CDW) [29].

### 3.8. Degradation of PHB Sheet

**Figure 4:**
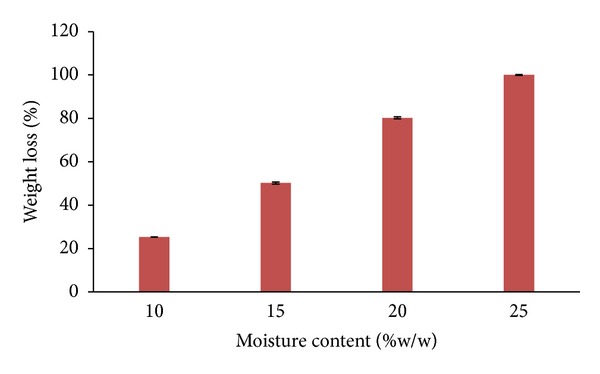
Effect of moisture content on PHB degradation in compost.

Polymers in natural environment are degraded through hydrolysis, mechanical, thermal, oxidative, and photochemical destruction, and biodegradation. One of the valuable properties of PHB is its biodegradability, and it was studied in simulated natural environment (in soil, compost, and industrial sludge) in laboratory. All test pieces of PHB starch blend sheet lost weight during incubation, but the degree of weight loss varied widely with environment in which the polymer sheets were incubated. The compost was found to be the better for degradation of PHB films compared to soil and industrial sludge. It is further supported by increase microbial load (5.0 × 10^6^ cfu/mL^−1^ to more than 3.0 × 10^9^ cfu mL^−1^/g) (Table 6) in compost after 30 days, where PHB could serve as nutrient source by microflora. The microbial population was increased in compost after 30 days. The bacteria detected on the degraded PHB films were dominated by the genera *Pseudomonas*, *Bacillus*, *Azospirillum*, *Mycobacterium*, *Streptomyces, Aspergillus*, and Penicillium [30]. The rate of degradation of the PHB films was 25.3% and 50.2% within 30 days in compost containing 10% and 15% moisture, respectively, while in the compost containing the 20% and 25% moisture the PHB films were found to be degraded into fine pieces in 30 days resulting into 80.2% and 100% loss in wt of PHB films (Figure 4) indicating the importantance of moisture content of the compost. Similar observation was noted by Wang et al. [11], Woolnough et al. [31], and Kulkarni et al. [10].

## 4. Conclusion 

The isolate *Bacillus subtilis* NG220 from sugarcane field area was able to efficiently utilize the sugar industry waste water as nutrient source for PHB production. The first line of impression of this study concludes that the sugar industry waste water could directly serve as an inexpensive nutrient source for production of biodegradable plastic. Thus, this study may solve the problem of costly treatment of sugar industry waste water as well as high production cost of biodegradable bioplastic and help in conservation of petroleum products which were used in the commercial production of plastic production.

## Figures and Tables

**Table 1:** Physiochemical characteristics of sugar industry waste water.

Parameter	Concentration (mg/L)
pH	4.2–6.9
Total solids	1,076 ± 414
Suspended solid	631 ± 227.4
COD	1,395 ± 317
BOD	1,300 ± 238
COD/BOD	1.6 ± 0.49
BOD/N/P	100 : 1.69 : 0.69
Nitrogen	11.48 ± 6.54
Phosphorous	4.36 ± 2.47

**Table 2:** Experimental culture conditions for optimization of various parameters under stationary condition of growth*.

Rigid parameter	Name of the parameter			
	Incubation period (h)	pH	Inoculum age (h)	Temperature (°C)
Incubation period (h)	Observed	7	18	30
pH	72	Observed	18	30
Inoculum age (h)	72	7	Observed	30
Temperature (°C)	72	7	18	Observed

*Production medium containing treated undiluted sugar industry waste water.

**Table 3:** Morphological and biochemical characteristics of isolate NG220.

	Observation
Morphological characters	
Elevation	Umbonate
Edge	Entire with undulate
Internal character	Rough
Colour	Creamy off-white
Biochemical characters	
Gram reaction	Gram +ve rod
Glucose fermentation	+ve
Sucrose fermentation	+ve
Lactose fermentation	−ve
H_2_S production	−ve (no gas observed)
MR test	+ve
VP test	−ve
Catalase test	+ve

**Table 4:** Distance matrix analysis for homology (Kitmura-parameter-2) for isolate NG220.

*B. lichnif *	0									
*B. sp. BTB_C *	0	0								
*B. subtilis *	0.01	0.01	0							
*Query_NG220 *	0.01	0.01	0	0						
*B. amyloliq *	0.01	0.01	0	0	0					
*B. atropae *	0.02	0.02	0.01	0.01	0.01	0				
*B. pumilus A *	0.03	0.03	0.03	0.03	0.03	0.02	0			
*B. cereus AT *	0.06	0.06	0.06	0.06	0.06	0.06	0.06	0		
*B. thuringi *	0.06	0.06	0.06	0.06	0.06	0.06	0.06	0	0	
*B. Anthraci *	0.06	0.06	0.06	0.06	0.06	0.06	0.06	0	0	0
*B. coahuile *	0.06	0.06	0.06	0.06	0.06	0.06	0.06	0.04	0.04	0.04

**Table 5:** Growth and PHB production with sugar industry waste water.

% sugar industry waste water (v/v)	Biomass* (g/L)	PHB* (g/L)
20	0.48	—
40	0.66	—
60	0.82	—
80	4.42	0.012
100	10.211	4.928

*Biomass and PHB production after 72 h of incubation at 30°C with preprocessed sugar industry waste water.

**Table 6:** Degradation of polymer sheet in natural samples.

Natural sample	Incubation (days)	% weight loss of polymer sheet	Microbial population
Soil	0	0	3.0 × 10^6^
	10	30.2 ± 0.5	4.0 × 10^7^
	20	43.5 ± 1.2	4.9 × 10^7^
	30	70.6 ± 2.6	5.2 × 10^8^
	40	98.3 ± 2.1	5.4 × 10^8^

Compost	0	0	5 × 10^6^
	10	50.2 ± 0.5	2 × 10^7^
	20	80.2 ± 0.5	1.2 × 10^8^
	30	100%	3.0 × 10^9^

Industrial sludge	0	0	2.5 × 10^5^
	10	26.3 ± 1.2	3.2 × 10^6^
	20	57.6 ± 1.5	4.9 × 10^6^
	30	73.6 ± 1.5	5.6 × 10^6^
	40	97.2 ± 22	6.0 × 10^6^

*Degradation study was followed in lab conditions at room temperature.
